# Supplementary material for: Four millennia of long-term individual foraging site fidelity in a highly migratory marine predator
Source: Commun Biol. 2022 Apr 14;5:368. doi: 10.1038/s42003-022-03310-2 (PMC9010445; doi:10.1038/s42003-022-03310-2)
Supplement: Supplementary file 2 — Supplementary Materials [file 42003_2022_3310_MOESM2_ESM.pdf]

Supplementary Materials

Title: Four millennia of long-term individual foraging site fidelity in a highly migratory marine predator

Authors: Eric. J. Guiry[*1,2,3], Margaretta James[4], Christina Cheung[5], Thomas C.A. Royle[6]

Affiliations:

* Corresponding author

[1]School of Archaeology and Ancient History, University of Leicester, Mayor's Walk, Leicester, LE1 7RH, United Kingdom

[2]Department of Anthropology, Trent University, 1600 West Bank Drive, Peterborough, ON, K9L 0G2, Canada

[3]Department of Anthropology, University of BC, 6306 NW Marine Drive, Vancouver, BC, V6T 1Z1, Canada

[4]Land of Maquinna Cultural Society, Mowachaht/Muchalaht First Nation, Tsaxana (Gold River) BC, Canada

[5]Research Unit: Analytical, Environmental & Geo-Chemistry, Department of Chemistry, Vrije Universiteit Brussel, AMGC-WE-VUB, Pleinlaan 2, 1050, Brussels, Belgium

[6]Ancient DNA Laboratory, Department of Archaeology, Simon Fraser University, 8888 University Drive, Burnaby, BC, V5A 1S6, Canada

Supplementary Materials

Supplementary Figure 1. Boxplots showing estimated Bayesian standard ellipse areas (SEA_B) for each location. From dark to light, boxes show 50%, 75%, and 95% credible intervals. Red “x”s show the modes for each group.

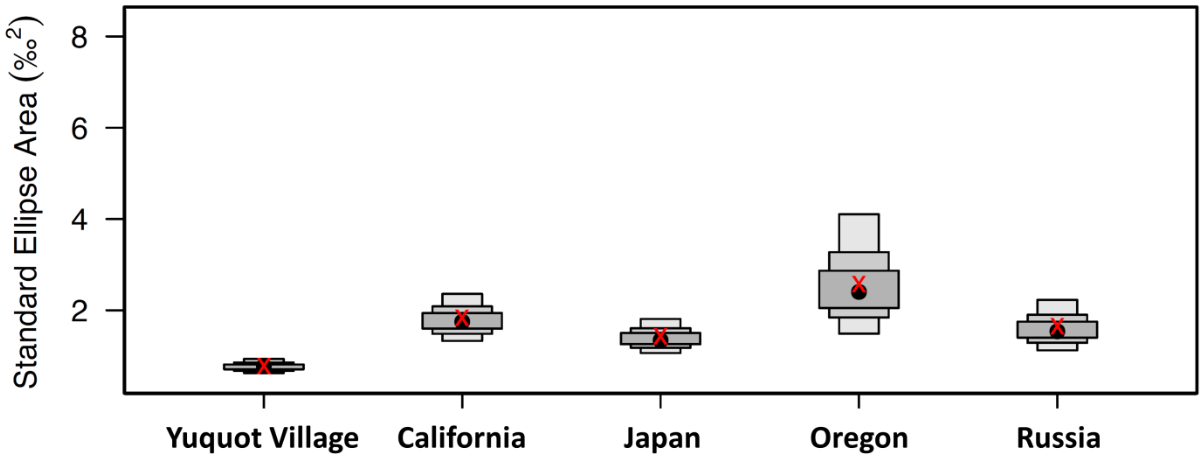

Supplementary Figure 2a. Results from bootstrapping simulation calculating standard deviations of 93 (the size of the samples from the Yuquot site) $\delta^{13}C$ values selected at random (1000 times) from across all regions (*n*=250). Red line shows standard deviation for the samples (0.39‰) from the Yuquot site.

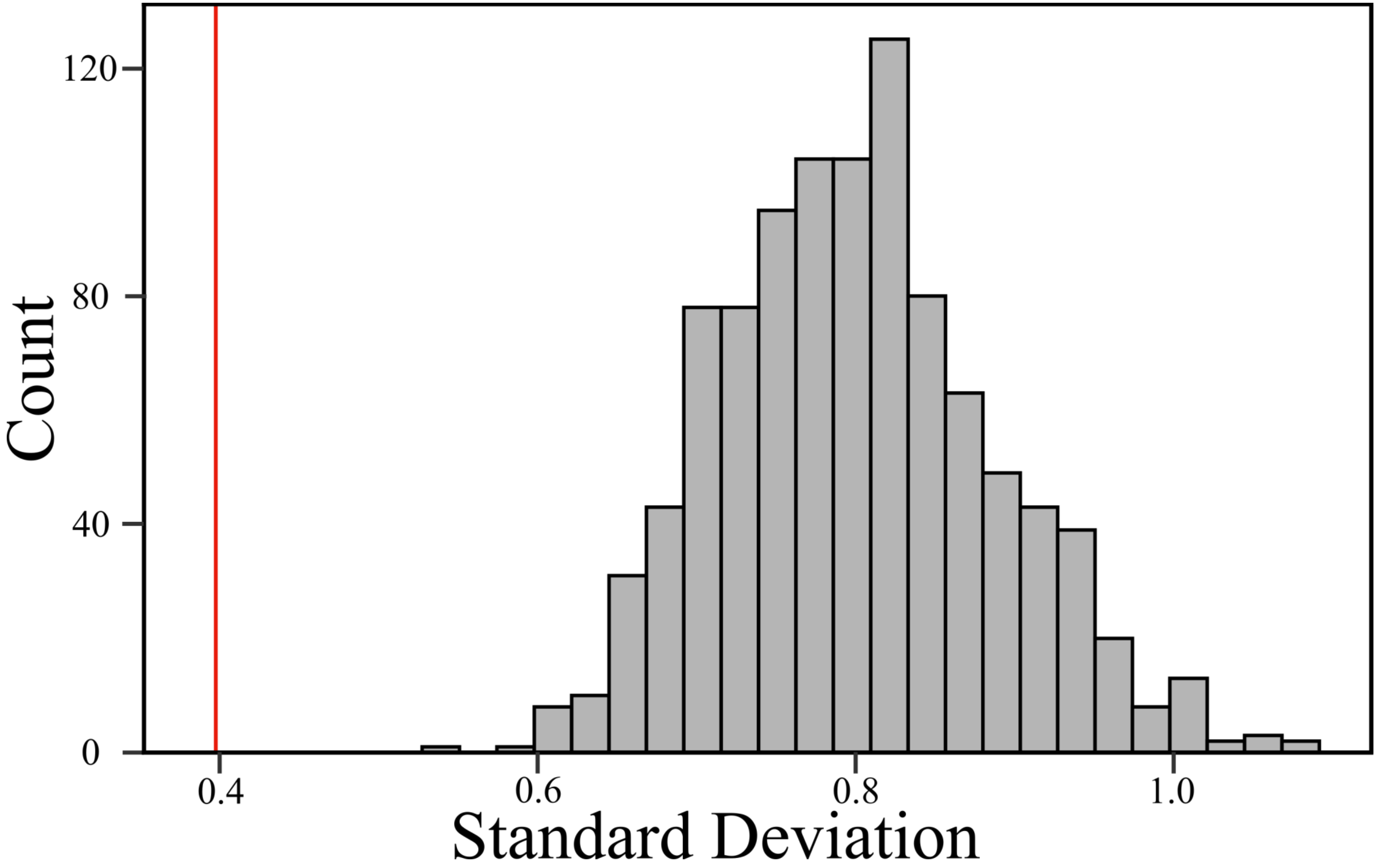

Supplementary Figure 2b. Results from bootstrapping simulation calculating standard deviations of 93 (the size of the samples from the Yuquot site) δ^{15}N values selected at random (1000 times) from across all regions (*n*=250). Red line shows standard deviation for the samples (0.68‰) from the Yuquot site.

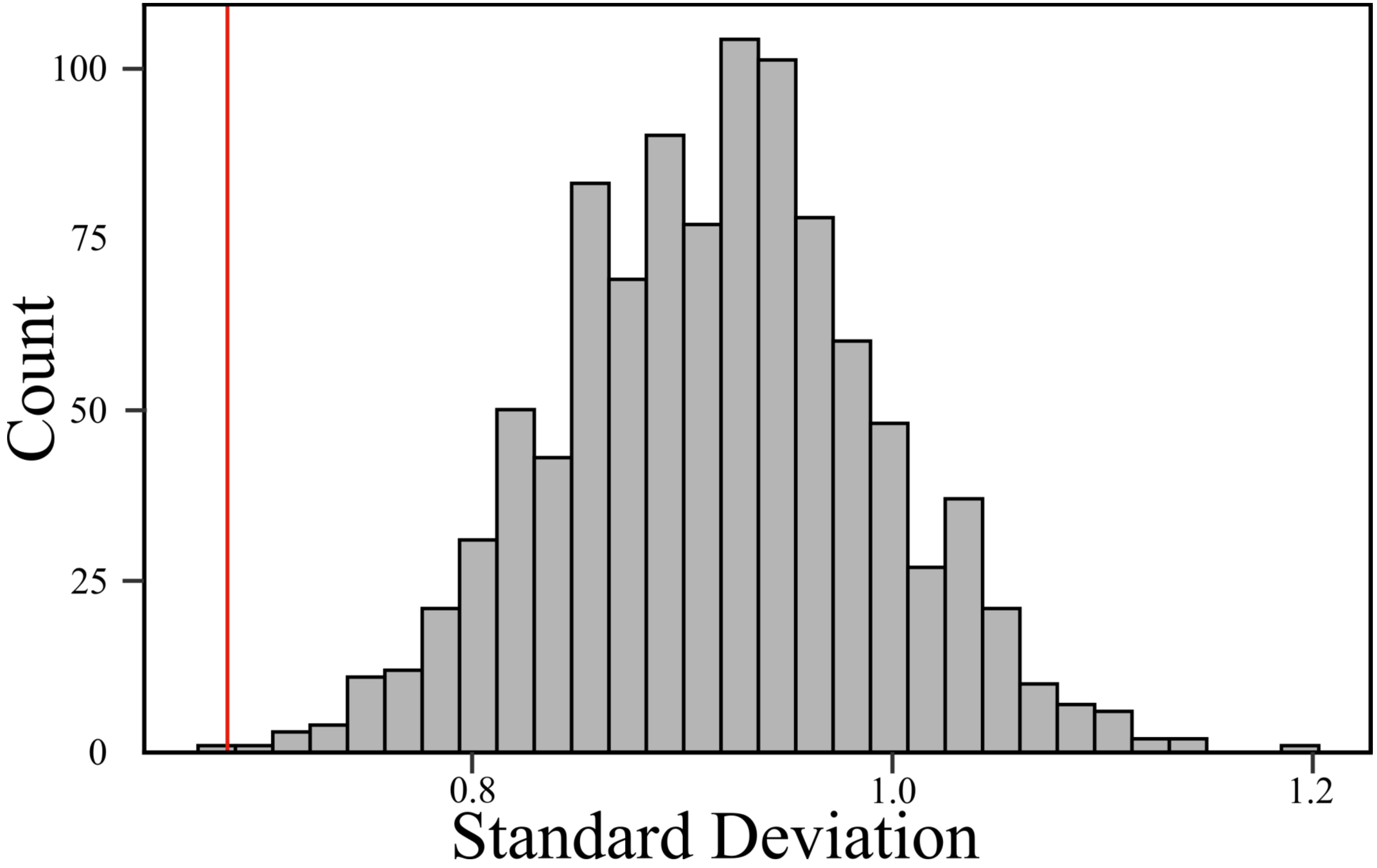

Supplementary Figure 3. Short-tailed albatross bone collagen (red circles) and feather (blue circles) isotopic compositions. Convex hulls are shown for bone (n=93; red solid line) and bone plus feather (n=95; blue dashed line) samples. Analytical uncertainty is shown in lower right.

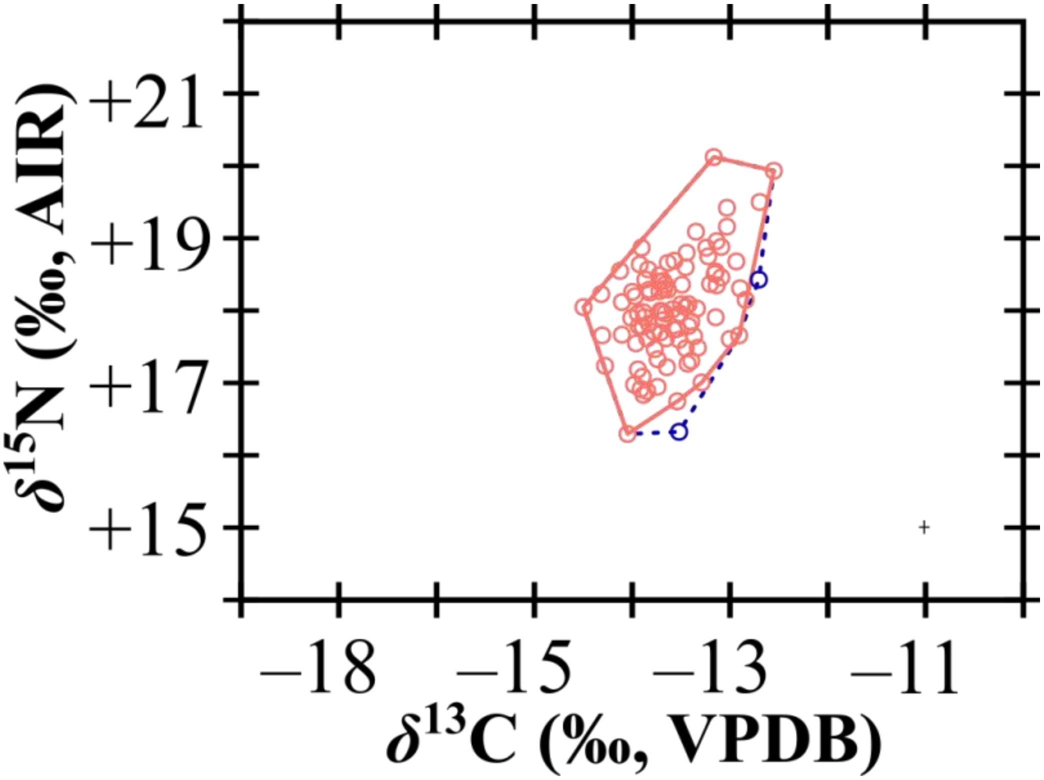

Supplementary Table 1. Mean and standard deviations for isotopic Yuquot short-tailed albatross compositions groups by bone.

**Bone**	***n*=**	**δ^{13}C (‰)**	**δ^{15}N (‰)**
Humerus	23	-14.4 ± 0.5	18.3 ± 0.7
Wing digit	25	-14.6 ± 0.4	17.7 ± 0.5
Coracoid	40	-14.6 ± 0.3	18.0 ± 0.7
Tarsometatarsus	5	-14.7 ± 0.3	18.5 ± 0.5

Supplementary Table 2. Results of Shapiro-Wilk test for all temporal zones. Bold indicates significant ($p \leq 0.05$) results.

	**Zone 1**		**Zone 2**		**Zone 3**		**Zone 4**	
	**δ^{13}C**	**δ^{15}N**	**δ^{13}C**	**δ^{15}N**	**δ^{13}C**	**δ^{15}N**	**δ^{13}C**	**δ^{15}N**
n=	5	5	25	25	39	39	24	24
W	0.823	0.971	0.977	0.972	0.964	0.968	0.971	0.908
p	0.123	0.881	0.819	0.686	0.237	0.329	0.702	**0.032**

Supplementary Table 3. Results from a Mann-Whitney *U* test with a Bonferroni correction comparing δ^{15}N among temporal zones. Bold indicates significant ($p \leq 0.05$) results.

	**Zone 2**		**Zone 3**		**Zone 4**	
	U	*p*	*U*	*p*	*U*	*p*
**Zone 1**	0.053	0.053	0.403	0.403	1.000	1.000
**Zone 2**			0.635	0.635	0.000	**0.004**
**Zone 3**					0.613	0.613

Supplementary Table 4. Accepted (calibration) and observed long-term (check) isotopic compositions and standard deviations (1σ) for standards used in this study.

Name	Material	Number	δ^{13}C (‰, VPDB)	δ^{15}N (‰, AIR)	Standard Type
USGS40	Glutamic acid	NA	−26.39	−4.52	Calibration standard[1]
USGS41a	Glutamic acid	NA	+36.55	+47.55	Calibration standard[2]
MET	Methionine	1609	−28.62±0.10	−5.04±0.14	Check standard
SRM-1	Caribou bone collagen	500	−19.39±0.09	+1.84±0.12	Check standard
SRM-14	Polar bear bone collagen	370	−13.67±0.08	+21.62±0.18	Check standard
SRM-15	Deer bone collagen	104	−26.88±0.05	+6.90±0.08	Check standard
SRM-16	Seal bone collagen	132	−14.81±0.10	+16.91±0.08	Check standard

Supplementary Table 5. Standard deviations for calibration standards for all analytical sessions.

**Analytical Session**	**Standard**	**Number**	**δ^{13}C (1σ)**	**δ^{15}N (1σ)**
CN17-33	USGS40	9	0.03	0.06
CN17-35	USGS40	9	0.04	0.03
CN17-36	USGS40	12	0.03	0.05
CN19-06	USGS40	9	0.05	0.09
CN17-33	USGS41a	8	0.05	0.17
CN17-35	USGS41a	8	0.04	0.22
CN17-36	USGS41a	9	0.07	0.18
CN19-06	USGS41a	8	0.07	0.22

Supplementary Table 6. Means and standard deviations for check standards for all analytical sessions.

**Standard**	**Analytical Session**	**Number**	**δ^{13}C (1σ)**	**δ^{15}N (1σ)**
MET	CN17-33	7	-28.60 ± 0.08	-5.00 ± 0.02
MET	CN17-35	7	-28.60 ± 0.05	-5.04 ± 0.05
MET	CN17-36	10	-28.62 ± 0.04	-5.07 ± 0.09
MET	CN19-06	6	-28.55 ± 0.07	-5.08 ± 0.17
SRM-1	CN17-33	6	-19.39 ± 0.05	1.83 ± 0.03
SRM-1	CN17-35	6	-19.38 ± 0.02	1.78 ± 0.09
SRM-1	CN17-36	6	-19.41 ± 0.03	1.79 ± 0.06
SRM-14	CN17-33	5	-13.67 ± 0.03	21.59 ± 0.04
SRM-14	CN17-35	5	-13.66 ± 0.05	21.62 ± 0.05
SRM-14	CN17-36	5	-13.64 ± 0.06	21.62 ± 0.12
SRM-15	CN19-06	6	-26.84 ± 0.02	6.96 ± 0.08
SRM-16	CN19-06	4	-14.81 ± 0.02	16.99 ± 0.15

Supplementary Table 7. Standard deviations for sample replicates from all analytical sessions.

**Sample**	**δ^{13}C (A)**	**δ^{13}C (B)**	**δ^{13}C (1σ)**	**δ^{15}N (A)**	**δ^{15}N (B)**	**δ^{15}N (1σ)**
IUBC-3918	-14.92	-14.86	-14.89 ± 0.04	18.86	18.86	18.86 ± 0.00
IUBC-3919	-14.77	-14.83	-14.80 ± 0.05	18.22	18.26	18.24 ± 0.03
IUBC-3920	-14.34	-14.28	-14.31 ± 0.05	18.97	19.19	19.08 ± 0.16
IUBC-3922	-14.42	-14.42	-14.42 ± 0.00	18.64	18.54	18.59 ± 0.07
IUBC-3926	-15.02	-14.96	-14.99 ± 0.04	18.16	18.33	18.25 ± 0.12
IUBC-3928	-14.43	-14.36	-14.40 ± 0.05	17.26	17.27	17.26 ± 0.01
IUBC-3929	-14.38	-14.35	-14.36 ± 0.02	17.80	17.89	17.84 ± 0.06
IUBC-3965	-14.50	-14.45	-14.47 ± 0.04	18.09	18.08	18.08 ± 0.01
IUBC-3966	-14.74	-14.77	-14.76 ± 0.02	17.71	17.69	17.70 ± 0.01
IUBC-3967	-13.95	-13.95	-13.95 ± 0.00	17.57	17.62	17.60 ± 0.04
IUBC-3968	-14.94	-14.91	-14.93 ± 0.02	17.22	17.13	17.18 ± 0.06
IUBC-3991	-14.15	-14.21	-14.18 ± 0.04	18.74	18.74	18.74 ± 0.00
IUBC-3992	-14.60	-14.73	-14.66 ± 0.09	18.48	18.31	18.40 ± 0.12
IUBC-3993	-14.86	-14.84	-14.85 ± 0.02	18.33	18.51	18.42 ± 0.12
IUBC-3994	-15.07	-15.02	-15.04 ± 0.04	16.32	16.26	16.29 ± 0.05
IUBC-3995	-14.80	-14.82	-14.81 ± 0.02	18.29	18.28	18.28 ± 0.00
IUBC-7019	-16.15	-16.04	-16.09 ± 0.07	16.29	16.35	16.32 ± 0.04
IUBC-7020	-15.25	-15.23	-15.24 ± 0.01	18.40	18.43	18.42 ± 0.02

Supplementary Materials References

1 Qi, H., Coplen, T. B., Geilmann, H., Brand, W. A. & Böhlke, J. Two new organic reference materials for δ^{13}C and δ^{15}N measurements and a new value for the δ^{13}C of NBS 22 oil. *Rap Com Mass Spectrometry* **17**, 2483-2487 (2003).

2 Qi, H. *et al.* A new organic reference material, l- glutamic acid, USGS41a, for δ^{13}C and δ^{15}N measurements − a replacement for USGS41. *Rap Com Mass Spectrometry* **30**, 859-866 (2016).

Supplementary Data 1 References

Eda, M. *et al.* Inferring the ancient population structure of the vulnerable albatross Phoebastria albatrus, combining ancient DNA, stable isotope, and morphometric analyses of archaeological samples. *Conserv. Genet.* **13**, 143-151 (2012).

Vokhshoori, N. L. *et al.* Broader foraging range of ancient short-tailed albatross populations into California coastal waters based on bulk tissue and amino acid isotope analysis. *Mar. Ecol. Prog. Ser.* **610**, 1-13 (2019).

Guiry, E. J. & Szpak, P. Improved quality control criteria for stable carbon and nitrogen isotope measurements of ancient bone collagen. *J Archaeol Sci* **132**, 105416, doi:https://doi.org/10.1016/j.jas.2021.105416 (2021).
