# Supplementary material for: Four millennia of long-term individual foraging site fidelity in a highly migratory marine predator
Source: Commun Biol. 2022 Apr 14;5:368. doi: 10.1038/s42003-022-03310-2 (PMC9010445; doi:10.1038/s42003-022-03310-2)
Supplement: Supplementary file 3 — Description of Additional Supplementary Files [file 42003_2022_3310_MOESM3_ESM.pdf]

Description of Additional Supplementary Files

File name: Supplementary Data 1.
Description: Isotopic and elemental compositions as well as available context information for all samples considered in this study. NR stands for “Not Reported” in cited study. RBCM denotes historical taxidermy specimens from the Royal British Columbia Museum. For source data from Vokhshoori et al. 2019, molecular C:N has been corrected to atomic C:N (i.e., multiplied by [14.007/12.011]). Bolded C:N ratios indicate sample did not pass collagen QC criteria (liberal criteria used – see Guiry and Szpak 2021). For full references, see Supplementary Materials file.
